# Supplementary material for: Are carbon emissions trading and green financial instruments synergistic? -Comprehensive quantitative research based on content analysis
Source: PLoS One. 2024 Mar 7;19(3):e0298601. doi: 10.1371/journal.pone.0298601 (PMC10919601; doi:10.1371/journal.pone.0298601)
Supplement: S1 Data — (ZIP) [file pone.0298601.s001.zip › Data/地级市Z_F工作报告.zip!/║■▒▒/╗╞╕╘╩╨/└┤╫╘╣1⁄2╓┌║┼í╛┬φ┐╦╩2╛▌═°í┐_2019.docx]

各位代表：

　　现在，我代表市人民政府向大会报告工作，请予审议，并请各位政协委员和其他列席人员提出意见。

　　一、2018年工作回顾

　　过去一年，在市委坚强领导下，我们坚持以习近平新时代中国特色社会主义思想为指导，深入贯彻党的十九大和习近平总书记视察湖北重要讲话精神，坚决落实省委、省政府和市委的决策部署，按照高质量发展要求，全市上下齐心协力、攻坚克难，积极应对经济下行，经济社会平稳健康发展。预计全市地区生产总值突破2000亿元，增长7.5%;规模以上工业增加值增长7.3 %;固定资产投资增长11%;一般公共预算收入增长9.5%;社会消费品零售总额增长11.3%;外贸出口增长16.5%;城乡居民人均可支配收入分别增长7.5%、8.5%。

　　经济结构持续优化。三次产业比20.22:39.49:40.29。工业经济扩量提质。规模以上工业企业达到1448家，年产值过亿元企业500家、过10亿元企业16家。市区3个百亿项目顺利推进，黄冈晨鸣南区30万吨浆粕生产线、绿宇环保20万吨环保面料生产线试产，星晖汽车完成厂房主体结构建设。实施技改项目230个，完成投资144亿元。黄冈伊利、科峰传动、科普达等入选省级隐形冠军企业名单，索菲亚家居建成首个工业4.0生产线。高新技术企业达到285家，高新技术产业增加值占GDP比重达8.5%。新能源新材料、智能制造、节能环保等产业快速发展,建筑业产值突破千亿大关。现代农业加快发展。“三品一标”农产品达到556个，规模以上农产品加工企业509家、实现产值620亿元，建成各类现代农业示范园区221个。能人回乡创业“千人计划”签约项目1313个，开工建设1214个，完成投资253.04亿元。成功举办首届农民丰收节。启动农业区域公用品牌创建，蕲艾跻身中国区域品牌地标产品百强，英山云雾茶获第二届国际茶博会金奖。现代服务业快速发展。接待游客数量3650万人次、旅游综合收入255亿元，新增4A景区2家，东坡赤壁、红安将军文化博览园等入选国省优选旅游项目，李时珍文化旅游区、鄂东禅文化旅游区、罗田“五景区五小镇”等加快推进。电子商务交易额突破800亿元，7个县市成为国家电子商务进农村示范县。推进商旅文体康融合发展，成功举办李时珍诞辰500周年系列活动，实施地标优品“五进一出”工程，“一会一节”协议购销金额122.87亿元。

　　四大行动持续发力。全市“双百”项目开工106个、完成投资262.56亿元，集创云天电池材料、新明珠绿色建材等17个省级重点项目全部开工入库。新落地PPP项目10个、总投资64.22亿元。新谋划重大项目589个、总投资4764亿元。招商引资再创新高，新引进亿元项目512个、协议投资2496亿元，新开工334个，新投产169个。大健康产业投洽会协议投资496.8亿元。深化“两月一单”，为813家企业解决要素保障问题，50家困难企业实现复工复产。新增市校合作高校2所，新聘科技副总132个，新建研发平台51家，转化科技成果94项。新发展市场主体7.75万户，五类市场主体达到53.1万户。

　　三大攻坚战持续推进。严控金融风险和政府债务风险，完善监测预警与化解机制，守住不发生区域性系统性风险底线。长江大保护九大行动、“双十”工程、雷霆行动十大专项整治成效明显，水、气、土污染防治强力推进，中央环保督察年度整改任务提前完成，河湖库长制全面施行，自然保护区管理加强，区域环境质量明显改善。“四个三重大生态工程”全面推进，建改农户厕所22.58万座，精准灭荒12.3万亩，106个乡镇生活污水处理项目基本建成，城乡生活垃圾无害化处理全面启动。脱贫攻坚取得决定性进展，有望实现罗田、英山、团风摘帽，141个贫困村出列,16.4万人脱贫。深化拓展产业扶贫，易地扶贫搬迁任务全面完成，健康扶贫、教育扶贫、政策兜底扶贫实现应扶尽扶。获评央视“2018年度魅力文旅扶贫城市”，闻彬军获全国脱贫攻坚奉献奖。红安脱贫摘帽的成功实践，为我们创造了经验、增添了信心，老区人民摆脱贫困、同步小康的愿景一定能够如期实现!

　　城乡面貌持续改观。启动城市总体规划修编，加快实施“一区两带”，城镇化率提高到47.12%。有序推进市区“双修”，新开工市政工程108个、完成投资7.3亿元，遗爱湖公园基本建成。大别山金融中心竣工，万达广场开业，市委党校新校区建成，资本大厦、市综合馆等项目加快推进，大别山医疗中心、市妇幼保健院搬迁进入倒计时，市疾控中心完成搬迁。完成交通投资142.6亿元，两条高铁、两座大桥、两条高速、长江645工程快速推进，提档升级农村公路2780公里，提前两年完成1万公里“455”安防工程。四项“补短板”重大工程提速，美丽乡村建设有序推进。完成水利投资56.5亿元，入江重要支流治理、病险水库除险加固、重点易涝地区外排能力建设加快推进。建成高标准农田75万亩，光网覆盖率实现100%，电网改造升级完成投资13.39亿元。黄冈大别山地质公园被联合国教科文组织批准为世界地质公园，黄州获评全国“四好农村路”示范县，英山成功创建“中国天然氧吧”。加快推进黄团浠交通一体化，巴河特大桥开工建设，南湖收费站撤除，全市普通公路政府还贷性收费全部取消。

　　改革红利持续释放。供给侧结构性改革有力推进，启动关改搬转化工企业36家，商品房去化周期控制在合理区间，政策性减税13.68亿元，降低用电成本1.08亿元。政务服务“一张网”实现省市县三级联通，取消、承接、下放行政审批事项47项，市政务服务中心启动整体搬迁，工业投资项目流程再造。农村集体产权制度改革有序推进，市农村综合产权交易中心挂牌运营。中央和省级改革试点成效明显，农垦和龙感湖农场改革、人才发展体制、黄冈高新区人事和薪酬制度、税管体制、国有企业等重点改革稳步推进，城管执法、县管校聘等改革试点成果扩大，小池省级全面深化改革示范区建设取得实效。推进金融创新，降低企业融资成本6.5亿元。新三板挂牌企业19家，四板挂牌企业500家，新增直接融资6.48亿元，祥云集团主板上市已报会，香江电器、远东股份IPO报辅。产业基金达到19支，总规模213亿元。对接湖北国际物流核心枢纽，启动航空物流园建设。跨江合作、环大别山省际协作纵深推进。

　　民生质量持续改善。财政支出八成以上投向民生领域。城镇新增就业7.36万人，2.4万人实现再就业，城镇登记失业率1.98%。社保提标扩面，社会救助标准提高，21.7万名困难群众享受城乡低保，5.65万名农村特困人员得到供养，2.38万人接受临时救助，20万贫困学生得到资助，12.9万残疾人获得两项补贴。城乡医保全市统一，县域医联体建设、基层医疗卫生综合改革深入推进，有效控制药价虚高和医疗费用不合理增长。新解决41.29万农村人口饮水安全，危房改造1.47万户，棚户区改造住房1.26万套。城乡义务教育一体化加快推进，圆满完成“全面改薄”任务，补充教师1741人。推进市区教育优质均衡发展，4所学校建成开学。实施文化小康建设三年行动，启动国家公共文化服务体系示范区创建。电影《马兰花开》全国公映。黄梅获评国家级“千年古县”。成功举办国际半程马拉松、中国大学生羽毛球锦标赛、挺进大别山系列赛事，全民健身活动精彩纷呈。安全生产形势平稳，食品药品监管水平提升，非洲猪瘟疫情得到控制。推进“七五”普法，完善“1343”维稳机制，扫黑除恶专项斗争取得阶段性成果，深化法治信访，平安法治黄冈建设成效明显。全国文明城市创建扎实推进。加强基层治理，村(居)民委员会换届全面完成。

　　自身建设持续加强。以党的政治建设为统领，树牢“四个意识”，坚定“四个自信”，践行“两个维护”，把真抓实干、埋头苦干作为转作风的有力武器，政府系统党的建设和作风建设不断加强。自觉接受人大监督、政协监督和社会监督，办理人大代表建议70件，政协提案308件。获评第五届“中国法治政府奖”提名奖。落实全面从严治党主体责任，严格执行中央八项规定及其实施细则精神，持之以恒正风肃纪，支持监察体制改革，强化审计监督，政府系统的执行力和公信力进一步提升。

　　国防动员、国家安全、人民防空、民兵预备役、优抚安置等工作进一步加强。与工会、共青团、妇联、工商联、科协、文联等群团组织联系进一步密切。对台、民族宗教、外事侨务、移民、农垦、老龄、统计、保密、档案、气象、水文、地震、方志、公积金管理、公共资源交易、新闻出版、广播电视、机关事务管理、慈善事业、残疾人事业、妇女儿童事业等都取得新成绩。

　　各位代表!过去一年，我们全面贯彻党中央决策部署和省委、市委工作要求，顶住转型阵痛的巨大压力，经受“三期叠加”的严峻考验，在困难和挑战中砥砺前行，每一项成绩都凝结着广大干部群众的智慧和汗水。在此，我代表市人民政府，向全市人民，向人大代表、政协委员，向各民主党派、工商联、无党派人士、人民团体、离退休老同志，向驻黄部队、武警官兵、政法干警、部省在黄单位，向所有关心支持黄冈发展的同志们、朋友们，致以崇高的敬意和衷心的感谢!

　　我们清醒地认识到，前进的路上不会一帆风顺。我市仍处于深度转型阶段，周期性和结构性矛盾交织，经济下行压力较大，发展韧性、动能接续、创新能力有待加强;工业投资占比较低，民间投资意愿不强，重大项目支撑不足;实体经济仍然比较困难，融资难融资贵问题依然突出;三大攻坚战任务艰巨，民生领域欠账较多，决胜全面小康还有诸多短板;营商环境还不优，政府的执行能力、服务意识、工作水平有待进一步提高，少数部门和干部担当精神不强，不作为的现象不同程度存在。我们一定正视困难和问题，采取坚决措施加以解决。

　　二、2019年工作建议

　　2019年是新中国成立70周年，是全面建成小康社会关键之年，是推动新时代高质量发展的奋斗之年。新的一年，新的希望。我国发展仍处于重要战略机遇期，经济长期向好的态势没有改变。在全省“一芯两带三区”战略布局中，黄冈处于“芯之瓣、带之翼、区之核”，战略地位更加突出。我们正处于厚积薄发、加速崛起的新阶段，政策利好在叠加，要素资源在集聚，经济走势在回升，发展短板在破解，有利于我们抢抓机遇，快速发展。同时，外部环境稳中有变，区域竞争激烈，自身跨越关口、转型攻坚，也将带来严峻挑战。我们必须敢字当头、敢于争先，坚持高质量发展导向，突出“双强双兴”，推进“四大行动”，加快老区振兴崛起，决胜全面小康，建设全省区域性增长极。

　　今年政府工作的总体思路是：以习近平新时代中国特色社会主义思想为指导，全面贯彻党的十九大和十九届二中、三中全会及中央经济工作会议精神，深入落实习近平总书记视察湖北重要讲话精神，统筹推进“五位一体”总体布局，协调推进“四个全面”战略布局，坚持稳中求进工作总基调，坚持新发展理念，坚持推动高质量发展，坚持以供给侧结构性改革为主线，坚持深化市场化改革、扩大高水平开放，加快建设现代化经济体系，推动三大变革，继续打好三大攻坚战，落实“六稳”工作部署，统筹推进“稳促调惠防”各项工作，在全省“一芯两带三区”区域和产业战略布局中找定位、谋突破，在新时代高质量发展中力争上游，为全面建成小康社会收官打下决定性基础，以优异成绩向新中国成立70周年献礼。

　　主要预期目标是：地区生产总值增长7.5%左右，规模以上工业增加值增长7.5%左右，固定资产投资增长10.5%左右，社会消费品零售总额增长10.5%以上，一般公共预算收入增长8%，城乡居民人均可支配收入增长8%以上，城镇登记失业率控制在 4.5%以内，全面完成省下达的节能减排任务。

　　围绕上述目标任务，重点做好八个方面的工作：

　　(一)做强经济支撑，在稳增长上力争上游。突出投资的关键性作用，发挥消费的基础性作用，增强环境的保障性作用，加速要素聚集，做强经济支撑。

　　突出项目建设。完善项目生成机制，打通项目快速落地“绿色通道”。建好项目储备库。围绕长江经济带绿色发展、大别山振兴发展、新型城镇化、乡村振兴、产业转型升级等重大战略，谋划一批重大项目，新增储备库项目总投资4000亿元以上。聚焦脱贫攻坚、铁路、水利、能源、生态环保、社会民生等重点领域，积极申报中央和省预算内投资项目，争取资金不少于40亿元。积极争取土储债、公路债、棚改债等政府专项债券。推进燕矶长江大桥、沿江高铁、蕲太东、黄团浠水资源配置工程、西隔堤加固等项目前期工作。建好项目建设库。加快黄冈晨鸣、中粮肉食、中船712所电池、红安新型建材产业园、燕加隆产业园、武穴电子信息产业园、百恒科技纺织产业园、龙感湖绿色纺织面料基地等产业项目建设，加快黄黄高铁、合安九高铁、武穴长江大桥、棋盘洲长江大桥、江北铁路及长河综合治理等重大基础设施项目建设。建好项目达效库。推进绿宇环保一期、星晖汽车一期、顾家家居、大别山电厂二期、招云寨风电等项目建成投产。

　　突出招商引资。新引进亿元项目350个以上，协议投资2500亿元以上，资金到位率40%以上。强化产业链招商。围绕传统支柱产业和战略性新兴产业，开展主题招商。围绕市区三个百亿企业和龙头企业，引进上下游项目。强化招大引强。紧盯大企业、大集团战略意向，大力引进央企国企、知名民企和“独角兽”企业。强化平台招商。加强与各异地商会、黄冈师院和黄冈中学校友会等联系，深化乡情友情招商、商协会招商，扩大以商招商。开展京津冀、长三角、珠三角、中部地区等系列招商活动。实行招商引资“一月一调度、双月一活动、季度一排名”，高频率推进。落实招商政策，兑现政府承诺，以政府诚信带动社会诚信，让诚信成为黄冈的靓丽名片!

　　突出消费提升。顺应消费升级，大力发展文体康养游等幸福产业，促进吃穿用住行消费提档升级，扩大教育、医疗、家政等服务消费。举办第二届大别山(黄冈)地标优品博览会暨文化美食节，完善地标优品目录管理，扩充“名特优”产品，做强产业联盟。积极发展商业新模式、新业态，健全城乡流通网络和售后服务体系，发展“线上+线下、商品+服务、零售+体验”，促进消费提质扩容。改善消费环境，规范市场秩序。落实个税专项附加扣除等政策，增强消费能力。因城施策、分类指导，构建房地产市场健康发展长效机制。

　　突出营商环境优化。最大限度减量提质、减时增效，建立公平透明开放的市场规则，打造法治化营商环境。企业开办时间压缩至3个工作日内，投资项目审批实现“三级八同”，工业项目实行联合勘察、联合图审、联合验收、50个工作日内取得施工许可。推进减证便民，创建“无证明城市”。开展“千名干部进千企”服务行动，帮助企业解决实际困难。

　　突出民营经济壮大。清理准入门槛规制，消除民企在准入许可、经营运行、招投标等方面的不公平待遇。清理惠企政策，全面落实更大规模减税降费政策。清理政府部门及平台公司拖欠民营企业账款，限时还账清零。清理垄断中介，注重市场培育，优化服务平台。清理企业难题，完善各级领导联系重点民营企业制度，加大对中小微企业和“双创”企业的金融支持，为民企赋能。构建“亲”“清”政商关系，健全企业家参与涉企政策制定机制。依法保护民营企业家合法权益和人身财产安全，以实际行动推动民营经济发展壮大，让民营企业家轻装上阵、大展宏图!

　　(二)加快新旧动能转换，在产业转型升级上力争上游。坚持扩量与提质、特色与创新并举，聚焦实体经济，推进转型升级，提高产业竞争力，着力构建现代化产业体系，在鄂东转型发展示范区中走在前列。

　　制造业高质量发展。建立重点产业“十个一”工程措施，加快发展先进制造业，着力构建现代工业产业体系。推动传统产业改造提升。聚焦食品饮料、纺织服装、医药化工、建筑建材、机械电子等传统支柱产业，重点实施106个万企万亿技改项目。引导建筑企业提档升级，大力发展装配式建筑和绿色建筑，支持团风、英山率先发展。推动新兴产业发展壮大。深度融入“一芯”、长江绿色经济和创新驱动发展带，发展智能制造、集成电路、生物产业、新能源与新材料等战略性新兴产业。大力发展“专精特新”企业，新增一批隐形冠军示范企业。实施骨干企业培育工程，支持龙头企业做大做强。实施中小企业成长工程，精准推动“个转企、小进规”，新增进规企业50家。推动品牌质量提升。支持企业增品种、提品质、创品牌，支持产业园区、特色产业集群争创“知名品牌创建示范区”。鼓励企业开展商标国际注册、产品国际认证。推动跨界融合。推进先进制造业与现代服务业深度融合，实施“万企上云”工程，新增省级“两化”融合试点企业10家。大力发展军民融合产业，创建军民融合创新示范区。

　　服务业提速升级。围绕10个服务业重点产业，健全保障机制，做大潜力产业。提升文旅产业。加强全域规划、资源、线路和营销统筹，推进城市旅游提质、乡村旅游提升、景区旅游提标，讲好黄冈故事，丰富旅游产品，推动旅游质量变革。支持麻城、罗田、红安、英山国家级全域旅游示范区创建，争取龟峰山5A景区创建验收，推进红安革命纪念地5A景区和蕲春国家中医药健康旅游示范区创建，举办首届大别山(黄冈)世界旅游博览会暨文化旅游推介活动。提升康养产业。抢占大健康产业蓝海市场，创新健康产业新模式，继续举办大健康产业投洽会，加快建设“中国•黄冈中医药健康谷”，打造“康养福地”品牌。提升物流产业。加快发展临空、临港、铁路物流和冷链物流。优化黄冈港经贸服务和流通功能，发展临港产业，建设临港新城。提升电商产业。培育本土电商龙头，深化与知名电商合作。持续推进商旅文体康深度融合，加快发展大文旅、大健康、大物流、大会展等现代服务业。

　　园区提档升级。加快国家级高新区创新发展，完善“一区多园”推进机制，统筹产业布局、功能分区、服务配套，构建核心引领、多点支撑、错位发展的新格局，实现园区对工业经济增长贡献率提高3～5个百分点。深化体制机制改革，扩大园区自主权，支持省级经济开发区扩区调区。推行区域性统一评价、“多规合一”“标准地”出让，开展先建后验试点。拓展高新区核心区发展空间，提高承载能力。深化与东湖高新区合作，加快光谷黄冈科技产业园建设。推进黄冈产业园重点项目建设，实现提质发展。加快白潭湖“四区一城”建设，对接顺丰空港城，启动“四大中心”项目。推进国土资源节约集约示范市创建，加大闲置土地清理处置力度，加快腾笼换鸟，推进“满园行动”。

　　县域经济进位升级。坚持市区一体，高昂市区龙头，注重分类指导，支持县域经济特色发展。实施“六个一”计划，每个县(市、区)培育一个百亿主导产业、谋划一个百亿潜力产业、打造一个百亿园区、引进一个十亿元制造业项目、新增一批规上工业、实施一个亿元以上PPP项目，力争县县进位，1～2个进入全省县域经济20强。进一步完善政策措施，激励各地在全省同类县(市、区)中找坐标、找差距，点燃激情比着干、敢为人先拼着干，拼出不胜不休的精气神，干出高质量发展的新业绩!

　　(三)深化改革开放，在创新发展上力争上游。坚持以改革释放生产力，以创新迸发创造力，以开放增强竞争力，激发市场主体活力，形成高质量发展的强大动能。

　　向改革要红利。深化市场化改革，全面落实市场准入负面清单制度。深化“互联网+放管服”改革，推行线上“一网覆盖”、线下“只进一门”、现场办理“只跑一次”。深化综合行政执法体制改革，推进商事制度改革，深化“四多合一”“证照分离”“照后减证”，加强“双随机一公开”监管。深化财税体制改革，推进全口径预算管理，深化增值税等税制改革。全面推进市县机构改革。推进大别山金融工程升级，深化新型政银担合作，发展小微金融、科技金融和普惠金融，保持信贷增长全省领先。设立大别山产业基金，发展各类产业投资基金。拓展股权融资和债券融资，扩大直接融资。推进中央和省级各项改革试点，深化国企改革、事业单位分类改革、环保垂管体制等改革。

　　向开放要空间。深化与武汉的融合对接，承接功能疏解和产业转移。融入长江经济带和中游城市群，承接沿海产业转移，深化港口合作、城市合作。推进小池、散花跨江合作，启动小池绿色发展三年计划。深化环大别山区域合作，合力推动大别山革命老区振兴发展。对接湖北国际物流核心枢纽，融入国家级空港经济综合试验区，规划建设黄冈临空经济区。加快推进黄州一类水运口岸建设。大力发展外贸新业态，引导黄冈外贸企业“出口回归”，支持广济药业、宏源药业等重点出口企业发展，推进生物医药、茶叶、新型建材等出口基地建设。积极参加国内外展会，全力开拓“两个市场”。扩大利用外资，提升国际化水平。

　　向创新要动力。完善以企业为主体、市场为导向、产学研相结合的技术创新体系，增强科技对经济发展的带动能力。新增高新技术企业40家，引进科技副总100个，R&D经费支出占GDP比重达到1%。大力实施“人才强市”战略，深化市校合作、千企联百校，深入推进大别山英才计划和“黄冈工匠”培养行动，加快引进一批高层次专业人才和科技领军人才。

　　(四)推进“三农”发展，在乡村振兴上力争上游。坚持农业农村优先发展，深入推进“五个振兴”，让黄冈农业强起来、农村美起来、农民富起来。

　　发展现代农业。深化农业供给侧结构性改革，实施“优质粮食工程”，稳定粮食生产，发展“虾稻共作、稻渔种养”“粮经饲”统筹，大力培育十大特色优势产业。打造都市农业示范区，创建一批现代农业产业园，建设一批精品果园、生态茶园、放心菜园、道地药园、美丽花园。支持龙感湖创建国家级现代农业示范区。实施农产品加工业提升行动，做强优质粮油、食用菌、中药材等特色产业集群。实施农业科技工程，提高农业机械化、信息化水平。实施品牌强农工程，加快区域公用品牌建设，完善标准化生产和质量监管体系。加快发展农业“新六产”，延长产业链、提升价值链、完善利益链，促进农业增效、农民增收。加强重大动植物疫病防控，发展绿色生态养殖。

　　深化农村改革。深化农村土地制度改革，完善承包地“三权分置”，探索宅基地“三权分置”办法。推进农村集体产权制度改革，全面完成清产核资和清人分类，盘活集体资源资产，多途径壮大村集体经济，基本消除村级集体经济空壳村、薄弱村。建成市县一体农村综合产权市场交易体系。引导农村土地有序流转，开展土地经营权入股发展农业产业化试点。

　　培育“三创”人才。强化人才集聚效应，实现乡村发展人力资本的整体提升。大力培育创富人才，实施新型职业农民和新型市场主体培育工程，支持龙头企业、家庭农场、农民合作社发展壮大，创办农业产业化联合体，完善农业社会化服务体系，带动小农户融入现代农业发展。大力培育创业人才，持续推进能人回乡创业“千人计划”，全面推行村级产业指导员、电商信息员和金融信贷员制度。大力培育创新人才，实施“一村多名大学生”计划，培养一支懂农业、爱农村、爱农民的“三农”工作队伍。

　　建设美丽乡村。聚焦“一带一片”，外修新村，内修新风，形成连线成片的美丽乡村集中区。全面推进农村人居环境整治，突出“三治一改”，提升村容村貌，打造一批美丽村落、美丽庭院、美丽田园。持续实施四项“补短板”重大工程，新建20万亩高标准农田，完成水利补短板年度任务，实现乡镇生活污水治理全覆盖，推进“四好农村路”示范创建，提档升级农村公路2000公里。支持浠水全国“四好农村路”示范县创建。完成4878户危房改造，实施农村饮水安全巩固提升工程。创新乡村治理，塑造文明乡风、良好家风、淳朴民风，传承和保护厚重的人文传统。

　　(五)统筹城乡一体，在新型城镇化上力争上游。推进以人为核心的新型城镇化，做优城市品质，提升乡村形象，加快城乡融合，建设美丽黄冈。

　　完善市区功能。深入实施城市建设绿色发展三年行动，提升市域中心城市品质。实施城市更新工程。持续推进棚户区和老旧小区改造，完善公共设施，打造15分钟社区生活圈。推进公共停车场建设、老旧小区加装电梯，规范小区物业管理。老城区800米、新区500米实现公厕全覆盖。启动市区备用水源建设，加强污水收集处理，提高城区防洪排涝能力。实施城市亮化和景观建设工程。加快赤壁公园和黄冈西站改造提升，增加城市绿量，打造长河等节点景观。实施智慧交通工程。加强道路改造和智慧交通建设，优化公交服务，提高通行效率。实施精细管理工程。加强市容市貌管理，深入推进全国文明城市创建。加快黄团浠一体化发展，坚持基础设施先导、民生一体共享，启动一体化供水和园区共建。

　　加快县城提质。坚持产城融合，完善功能，强化管理，提升水平。推进县市规划修编，深入开展文明城市、卫生城市、园林城市创建，提升县城品质。推进华夏幸福团风产业新城、麻城滨河新区、英山城西新区、浠水一河两岸、蕲春城南新区、武穴武山湖湿地慢城、黄梅高铁新区建设。

　　推进城乡融合。坚持全域规划，强化规划管控，实现“多规合一”。升级“一区两带”战略布局，深化临江四城绿色开发，培育一批工业强镇、商贸重镇、旅游名镇。加快公共服务和基础设施向农村延伸，推动城乡公交、供气、水电一体化，实现村级规划编制全覆盖。引导农村人口向城镇转移，帮助农村转移人口更好融入城市。

　　(六)决胜全面小康，在打好三大攻坚战上力争上游。三大攻坚战，事关决胜全面小康大局，必须精准施策，打好重点战役。

　　坚持底线思维，打好防范化解重大风险攻坚战。强化地方政府债务预算管理、限额管理，规范推进政府购买服务、政府和社会资本合作，积极稳妥化解存量债务。深入开展互联网金融风险专项整治，加强对类金融机构薄弱环节监管，严厉打击恶意逃废债务和非法集资、金融诈骗。加快政府融资平台公司市场化转型。强化社会风险防控，确保“六个不发生”。

　　坚持尽锐出战，打好精准脱贫攻坚战。今年是脱贫攻坚战三年行动的决战决胜之年。要对标“两不愁三保障”“三率一度”和“三落实”标准，全面完成剩余16.09万贫困人口脱贫、129个贫困村出列、蕲春和麻城摘帽任务。要精准施策，落实“七个到户到人”，做实“五位一体”产业扶贫，统筹健康扶贫、易地扶贫搬迁、教育扶贫、社会保障兜底等政策落地生根。坚持摘帽不摘责任、不摘政策、不摘帮扶、不摘监管，巩固脱贫成果。要全域发力，统筹贫困县与非贫困县、贫困村与非贫困村，防止两级分化、工作失衡。坚持志智双扶，注重典型引路，汇聚脱贫攻坚正能量。坚持政府、市场和社会互动，构建大扶贫格局。严格落实脱贫攻坚作风建设“十不准”规定，强化资金监管，坚决防止弄虚作假和消极腐败行为。

　　坚持绿色发展，打好污染防治攻坚战。坚决抓好中央和省级环保督察反馈问题整改，确保生态环境质量持续改善。推动长江经济带绿色发展，强化“三线一单”硬约束，巩固提升系列专项整治成果。深入推进精准灭荒和长江岸线造林绿化工程，加大自然保护区、湿地公园保护力度，推进白莲河生态保护与绿色发展示范区建设。深入推进大气、水、土壤污染防治三年行动计划，坚持源头防治，强化监测，联防联控，坚决打赢蓝天、碧水、净土保卫战。大力推进“双十”工程和新一轮雷霆行动，落实河湖库长制，严格水源地保护，大力整治黑臭水体和断面水质，守护一江清水出湖北。持续推进生态市四级联创，积极创建国家级生态文明建设示范区。推进污染源普查和领导干部自然资源资产离任审计。坚持统筹兼顾，既铁腕治污，又不搞“一刀切”。我们要倍加呵护宝贵的山水资源，用生态修复和环境保护留住绿水青山，用绿色发展赢得金山银山!

　　(七)增进百姓福祉，在民生改善上力争上游。民生工作无小事。既要量力而行，更要尽力而为，不断增强人民群众的获得感、幸福感、安全感。

　　提高社会保障水平。实施就业优先政策，以创业带动就业。加大企业稳岗支持力度，加强高校毕业生、农民工、退役军人等群体就业帮扶。保基本兜底线，实现城乡低保按标施保，应保尽保。保证养老金按时足额发放，提升养老机构服务质量。推进全民参保计划，完善城乡居民基本医疗保险、大病保险、医疗救助和异地就医联网直接结算制度。健全社会福利、慈善、法律援助等制度，加强农村留守儿童、妇女、老人和残疾人关爱服务。完善劳动争议处理机制，保障农民工工资支付，构建和谐劳动关系。

　　办好公平优质教育。实施学前教育三年行动计划，扩大普惠性资源供给。深化城乡义务教育一体化改革，加强乡村小规模和寄宿制学校建设，促进优质均衡发展。优化市区学校布局，加快消除“择校热、大班额”。深化高考综合改革，加快职业教育产教融合，支持高等教育内涵式发展，打造一批优质学校、特色专业。加强新时代教师队伍建设，保障教师待遇，提升教师素质，让每个孩子都能健康快乐成长!

　　发展卫生健康事业。推进健康黄冈十大行动，强化“三医联动”，全面深化医改，加强县域医联体建设，持续改善基层医疗服务，推进医疗人才队伍和信息化建设，深化爱国卫生运动，提升优生优育水平，防控重大疾病，维护和保障群众健康。加大国家“学生饮用奶计划”推广力度。办好市五运会及系列体育赛事，推进全民健身“六边工程”，打造运动之城、健康之城。

　　大力推进文化建设。坚定文化自信，培育和践行社会主义核心价值观。深入实施文化小康建设三年行动和国家公共文化服务体系示范区创建。加强县级融媒体中心建设，加快基层“五馆三场”和综合服务中心建设，推进文化惠民。实施优秀传统文化传承和文艺精品创作工程，加强革命文物保护和文化遗产保护传承。健全现代文化产业和市场体系，培育新型文化业态，争创国家级文化产业示范园区和示范基地。

　　加强和创新社会治理。学习“枫桥经验”，坚持“三治融合”，构建共建共治共享的社会治理新格局。加大全民普法力度，弘扬社会主义法治文化。深入推进扫黑除恶专项斗争，加强互联网安全管理，推进“雪亮工程”，发展智慧警务，完善社会治安防控体系，依法管理宗教事务，建设平安法治黄冈。推进“三合一”信访矛盾调解和律师“三进”，加快信访法治化改革。强化食品药品全过程、全链条监管，压实安全生产责任，确保人民生命财产安全。

　　依法加强国防动员和民兵预备役建设，深入开展国防教育和双拥共建，支持军队改革，支持驻黄部队建设，加强人民防空建设，加强退役军人事务管理和服务。做好地震、气象、档案、保密和方志等工作，做好第四次经济普查，支持工会、共青团、妇联等群团组织更好发挥作用。

　　(八)忠诚干净担当，在作风建设上力争上游。不忘初心，牢记使命，真抓实干，埋头苦干，用奋斗姿态交出让党和人民满意的答卷。

　　提升政治站位。把坚持党的全面领导作为最高政治原则，提高政府工作的政治站位。用习近平新时代中国特色社会主义思想武装头脑、指导实践、推动工作，不折不扣贯彻落实党中央方针政策和省委省政府、市委的决策部署。着力培养“五种思维”，提升“八个本领”，增强履职能力。

　　严格依法行政。改进政府立法工作，坚持规范公正文明执法，全面推进政务公开，加强法治政府建设。依法接受市人大及其常委会的工作监督和法律监督，自觉接受市政协民主监督，主动接受社会和舆论监督，广泛听取各民主党派、工商联、无党派人士和人民团体的意见。认真办理人大代表议案建议和政协提案，提高办理质效。

　　践行实干作风。从讲政治的高度狠抓落实、强化执行。力戒形式主义、官僚主义，讲短话、开短会、求实效。落实高质量发展重点工程专班推进机制，推进“互联网+项目化”管理，建立移动办公平台，实行“平台统一管理、线上线下同步、过程跟踪管控、实时预警督办、责任效果追溯”，以过程保证结果，以管理倒逼落实。

　　敢于担当作为。要增强敢字当头的政治定力，主动作为，在大力推动高质量发展中敢为人先。要敢于负责担当，遇到矛盾不回避，面对困难不退缩，履行责任不推诿。坚持尽职免责、失职追责，鼓励敢闯敢干、敢于斗争，严厉整肃庸政懒政怠政。大力容错纠错，关心基层干部，为担当者担当，为负责者负责，为实干者撑腰!

　　保持清正廉洁。坚决落实全面从严治党要求，严格履行主体责任和“一岗双责”，把纪律和规矩挺在前面，驰而不息整治“四风”。扎牢制度笼子，加强审计监督，规范权力运行，从源头上遏制腐败，以清廉作风赢得人民群众的信任和支持。

　　各位代表!长风破浪会有时，直挂云帆济沧海。让我们更加紧密地团结在以习近平同志为核心的党中央周围，树牢“四个意识”，坚定“四个自信”，坚决做到“两个维护”，在省委、省政府和市委的坚强领导下，锐意进取，敢为人先，力争上游，为奋力谱写黄冈高质量发展新篇章而努力奋斗!
